# Space, time and microdiversity: towards a resolution revolution in microbiomics

**DOI:** 10.1111/1758-2229.12897

**Published:** 2020-10-29

**Authors:** Lucas Paoli, Shinichi Sunagawa

**Affiliations:** ^1^ Department of Biology, Institute of Microbiology and Swiss Institute of Bioinformatics, ETH Zürich Zürich Switzerland

Predicting the future of our field can be an exciting and valuable thought experiment. For this exercise to be more than just wishful thinking, we set out to discuss some areas in environmental microbiology where we think current evidence suggests a particularly rapid change is taking place. To this end, through three imagined conversations, we explore some anticipated impacts on the current state of the field by what we refer to as a resolution revolution in microbiomics. We then conclude with a discussion of what in our view will help overcoming critical limitations in advancing our understanding of microbiome structure, function and dynamics in light of technical, analytical and conceptual developments.

*Ten years after graduating*, *a former PhD student and their advisor are reunited once more at a physical conference*, *an experience necessarily rare owing to environmental impacts*, *and sorely missed during recurring pandemic events. After catching up on years of personal news and gossip over dinner*, *the conversation inevitably turns to microbiome science…*

**Advisor:** So, thinking back, what were the main challenges that your research faced during those years in my lab?

**Former student:** Difficult to say really, but besides the constant struggle to keep up with the avalanche of microbiome papers being published every week… I would probably argue that most of the data available before the resolution revolution in microbiomics only gave a very coarse picture of the natural systems we were looking at. The limited *spatial resolution* available blurred our view of local heterogeneity, which was inevitably pooled or undersampled. Plus the limited *temporal resolution* only provided snapshots of the dynamic changes going on. *Phylogenomic resolution* was also a pain as it usually collapsed natural variation within and across microbial populations, restricting our understanding of strain‐level microdiversity. In the end, do you think that the field has actually managed to fully *resolve* all this, if you'll pardon the pun?

**Advisor:** I remember us speculating in a lab meeting once that a combination of robotics, microfluidics, single‐cell, gap‐free replicons sequencing would fix all of our problems. Actually, I think that the field has made huge leaps in most of these areas, although there is always room for improving resolution even further.

**Former student:** Definitely. I think the advances in data generation have changed the way we look at the sequence space, literally. We've moved away from unidimensional representations like FASTA files, towards the richer, graph‐based visualization of genomic variation. I'm actually quite happy about this: the graphs are a far better way to capture within‐species diversity and genome plasticity. I also suspect these changes in computational microbiomics were crucial in mainstreaming microdiversity.

**Advisor:** Absolutely! It's also been nice to see that this shift in data representation was accompanied by changes in lab‐based experiments. As we predicted back in the day, the shift in focus, back from descriptive, hypotheses‐generating data correlations to experimental hypothesis‐testing has genuinely begun to improve our mechanistic understanding of these systems. Still, even back then it was clear that tackling genes of unknown function and strain‐level differences would remain a challenge for quite some time.

**Former student:** I agree. But what do you think prompted this change?

**Advisor:** Well… arguably it all goes back to Baas Becking's tenet ‘*everything is everywhere*, but, *the environment selects*' and the many ways we have been able to use it as a working hypothesis. By adjusting the focus wheel, we would first define: What actually is a ‘thing’? Is it bacterial life? a microbial species? an eco‐evolutionary cohesive unit of genomic variants? an individual strain? a gene? an adaptive mutation? With haplotype‐resolved analyses, I feel we have made significant progress on some long‐standing questions. For instance: are conspecific strain population dynamics driven by growth and extinction of a standing stock of genomic variation, by the generation of new diversity, or by migration? How does the migration (or controlled addition/removal) of individual strains impact interactions within and functional properties of the whole community? I would say this promoted the value of microdiversity‐focused ‘N *plus* 1’ and ‘N *minus* 1’ types of experiments. In just these past few years, such experiments have shown how instrumental microdiversity is to understand microbiomes. For instance, the impact of ‘1’ on ‘N' and vice‐versa depends on the strain‐specific gene content of ‘1’ and ‘N', just as much as their species‐level composition. Of course, this has put a few noses out of joint by challenging the portability of results based on single lab‐grown strains to natural communities, since the model strains that many people worked with are often not the ones we find in nature, and natural communities are composed of diverse rather than clonal populations.

**Former student:** Very true. Along these lines, I think computational and experimental microbiomics is actually now progressing hand in hand pretty well. You cannot peer‐proof a computational analysis without functional validation anymore, the field just will not have it. Which is great for science, but… I feel this is complicating the process of me getting tenured!

**Advisor:** Right, because you think we had it easy?

**Former student:** Well actually… never mind. But coming back to the resolution revolution, I expected that it would have allowed us to better understand community functions, to predict how these would change in response to environmental and biotic changes, consequently to manipulate them, and eventually to inform decision makers. But it seems that there is still quite some way to go! We do have a few specific examples with a good enough mechanistic understanding to make robust predictions, but they remain the exception. Of course, the plethora of empiric data coupled with new AI tools now allow everyone to predict associations between microbiome features and phenotypes, particularly in the biomedical area. But knowing *that* it works is still seen by too many as more important than knowing *how* it works, so efforts to unravel the molecular mechanisms are sadly neglected. This does leave behind loads of black boxes for basic researchers to crack open and illuminate, of course. And on the upside, the microbiomics resolution revolution has paved the way to establish congruent ecological and evolutionary microbial units, which should allow the field to address how these affect microbiome structure, functions and dynamics, and this way further unite ecology and evolution.

**Advisor**: One thing that has not changed for sure though, is your focus on the conceptual and academic advances. But do not forget when completing that tenure application to describe how these advances in high‐resolution microbiomics are now impacting everyday lives!

*In a hospital*, *a patient suffering from what appears to be a gastrointestinal infection carefully listens to their doctor*, *who*, *based on results of tests conducted earlier the same day*, *is suggesting a new type of treatment…*

**Doctor:** If you recall, this morning I said we would need a current profile of your gut microbiome. To that end, we obtained a sample… upon induction of bowel movements… well, you know what I mean. Based on analysis of that sample, we now know the exact composition of your gut microbiome, not only what species and in what amount, but more specifically which variants within each species, and importantly the one causing the disease. This microbiome profile will be added to your unified medical records, where we already have your genome, immune profile and previous time points of your microbiome. It will allow us to take the most appropriate therapeutic decision for your specific situation.

**Patient**: Okay Doc. I read somewhere that a drug can lose its effect if you have certain bugs in your gut. Is that why you needed this information before deciding on a treatment?

**Doctor:** Correct. You can have drug‐bug or bug‐drug interactions, where either a drug will have a deleterious effect on your microbiome or your microbiome will prevent the drug from working properly. This is why much of modern medicine has moved away from a ‘one‐size‐fits‐all’ model. We now take microbiome‐informed therapy decisions based on a current picture of your microbiome. This is particularly relevant in your case. We now know that the pathogen causing your infection is resistant to most antibiotics and viruses that may target and kill it. But please do not worry. Fortunately the spread of antibiotic resistance during the past decades pushed the development of alternative methods and we now have several new options available for treating you, such as using close, non‐pathogenic relatives of the offending bacteria to out‐compete it and chase it out of your gut.

**Patient:** Does that mean that instead of a drug, the treatment you are suggesting is actually a microbe itself? Is that going to work?

**Doctor:** Yes, exactly. The chances for success of this approach are very good, but they do depend on several factors. Using the information we have on your immune system, as well as the composition of your microbiome, I browsed a strain database and selected the best candidate for the job. There are sometimes situations where this approach does not work out, for instance if the combination of a patient's immune and microbiome profile does not allow for a predictive outcome. But the science is now quite mature and allows us in many cases to restore a perturbed microbiome. And sometimes, AI uses the data collected from patients over the past couple of decades to inform us about therapeutic strategies, although we do not always know the exact mechanisms. These microbial strain‐mediated strategies are being used in more and more cases. For instance, oncologists have used select strains to stimulate the immune system during cancer immunotherapy.

**Patient:** Fascinating stuff! Although if you ask me, as long as it works, it does not matter how it works, does it?

*It's October 2031 and after the all‐time high summer temperatures a parent and their child are off to enjoy the school break on a picturesque but crowded beach on the English Channel. After walking past the recently renovated botanical garden, which now uses soil with a microbiome engineered to enhance the growth of exotic‐plants, they finally have sight of the sea. Just before reaching the pebbles, the child stops to read an information panel…*

**Child:** Hey, look! There is a map of the beach. Look, it also shows the quality of the water. That's so cool! So, I think here are the rivers and streams that go to the sea. They're green so it means good! Ah no, except this orange one. But maybe it's because of the sheep over there? Anyway, it says the water at the beach is excellent, so it's safe. Let us go swim!

**Parent**, *pulling out a mobile device from their bag*: Wait, come have a look at the augmented version of the map. You can see the water quality for every week over the last few years, and for several places along all of the rivers and streams on the map. Actually, this is pretty interesting. I remember being here before you were born and the old map was nothing like this. They did things quite differently. I think they would filter some water and specifically grow indicator gut bugs to see how much human waste was getting into the water. Look, here it says that with high‐throughput and high‐resolution sequencing strategies, they can now have detailed profiles with many more time points, and a spatial sampling. It says that they now know exactly, which strains of which species are dominating. I guess that's good because you can really tell the difference between bugs from the beach, animals or sewage.

**Child:** Hey, look! You can press that panel to see where the bugs come from. It says that by decoding their DNA, they can track the bacteria back to their source. Cool, most of the *E. coli* at the beach is not from humans and actually comes from the stream next to the meadow. There's also a bit coming from the water treatment plant upstream. I guess that makes sense.

**Parent:** Interesting, you'll have to tell your teacher about this after the holidays. Look, it also says that you can use similar microbial profiles to monitor air quality, although this is still just at the research stage. But for the water, they also have a second analysis: this one is about toxic blooms. What does it say?

**Child:** So, it says that naturally present algae and cyanobacteria can, under certain circumstances, form harmful blooms. When that happens we cannot go swimming where the bloom is. But based on the monitoring, they can predict when and where this will happen. It says that there is a 42.26% chance of a bloom starting on the other side of the beach in 2 weeks. So maybe that part will be closed soon?

**Parent:** I think so! It says they are testing a virus‐based method to control the blooms, but we better dive in while we still can!

**Child**, *running towards the water*: Way ahead of you!!!

For these imaginary conversations to become reality, and the field of microbiomics to advance as a whole, we find several challenges need to be overcome. In the case of the human gut, for instance, it is established that microbial communities undergo strong day‐to‐day compositional variations at the ‘species’ level (David *et al*. 2014), and that these ‘species’ harbour a relatively stable intra‐specific diversity (Schloissnig *et al*. 2013). This microdiversity is subject to both ecological and evolutionary processes (Garud *et al*. 2019; Zhao *et al*. 2019) and, as shown for other systems, it can affect the ‘species’ level composition of a community (Gómez *et al*. 2016; Padfield *et al*. 2020). However, answers to seemingly straightforward questions about this and other microbiomes remain elusive. For example, how many different genotypes of a given species (e.g., *E. coli*) does a gram of soil, an individual human, or a lake contain? What is the spatial structure, if any, of strain populations at the scales of micrometres (from epithelium to lumen) to meters (from oral cavity to rectum)? What are the abundance and gene expression dynamics within conspecific strain populations over time (e.g., from hourly to diurnal and seasonal variations in coastal waters)?

We argue that tackling such questions is currently limited by the operational resolution at which we look at microbial communities along **spatial**, **temporal** and **phylogenomic** dimensions. Advances along each one of these axes will contribute to a more holistic understanding of microbial communities and the intricate relationships between their members. Although such a holistic view may not be within reach yet, we believe that we are well on track and highlight some trends in technical, analytical and conceptual developments showing that we are in fact already amid a resolution revolution in microbiomics.

An increase in spatial and temporal resolution of microbiome composition largely relies on a more intensive sampling and sequencing effort, the latter of which is already being driven by dropping sequencing costs. Additional technological advances in low‐input DNA sequencing strategies (Rinke *et al*., 2016) combined with automated and remote sampling (Zhang *et al*., 2020) is likely to provide us with the ability to scale up the spatial and temporal frequency at which microbial communities are sampled, including at remote locations (Marx, 2020). The challenge of resolving within‐species genotypic diversity, or microdiversity, of microbiomes through metagenomics has taken the centre stage over the last few years (Yan *et al*., 2020; Van Rossum *et al*., 2020). We foresee that additional technological advances such as genome reconstruction from long‐read metagenomes (Moss *et al*., 2020; Beaulaurier *et al*., 2020; Kolmogorov *et al*., 2020) and high‐throughput single‐cell sequencing (Woyke*et al*., 2017) will provide the ground to further resolve population genotypic heterogeneity.

In addition to such technological developments, we anticipate the emergence of new strategies for data analysis and representation that need to be able to accommodate ongoing addition of new samples. For instance, in light of the increasing amount of sequencing data, graph‐based approaches represent an opportunity to rethink the mainstream data structures encoding genomic information (Mustafa *et al*., 2017; Karasikov *et al*., 2020). As we continuously uncover microbial microdiversity, leading to a steady growth of intra‐specific gene pools and information on genome‐scale structural variations, graphs emerge as an inevitable alternative for representing microbial pan‐genomes (Gautreau *et al*., 2020; Brown *et al*. 2020; Quince *et al*., 2020).

Conceptually, we anticipate these technological and analytical developments to prime a more mechanistic and predictive understanding of microbiomes. On the one hand, we expect the ever‐increasing amount of data to drive associations between microbiome read‐outs and disease state, treatment efficacy, ecosystem function or environmental factors (Sunagawa *et al*., 2015; Spanogiannopoulos *et al*., 2016; Wirbel *et al*., 2019). Such data‐driven analyses will keep generating more testable hypotheses on mechanistic causes and more powerful predictions. On the other hand, models of environmental and host‐associated microbial communities, including synthetic ones, and experimental strategies required to test mechanistic hypotheses are already emerging (Bai *et al*., 2015; Brugiroux *et al*., 2016; Bell, 2019). Notably, we expect a multidimensional increase in resolution to help researchers better grasp the complexity and diversity of natural microbial systems, and to influence environmental and health applications. Environmental monitoring strategies are almost certain to be impacted by advances in microbiomics approaches, which, in the case of bathing water quality assessment are already presented as viable alternatives to colony‐forming unit counts of coliforms (Council Directive 2006/7/CE, 2006; Tan *et al*., 2015). Furthermore, studies identifying strain‐level variation and diversity as drivers of community composition (Gómez *et al*., 2016; Padfield *et al*., 2020) and host physiology (e.g., cancer immunotherapy, Fluckiger *et al*., 2020) exemplify the importance of resolving microdiversity for future translational applications.

Although our focus here was set on nucleic acid sequencing‐based omics, other emerging technologies, including meta‐proteomics, meta‐metabolomics and high‐throughput imaging (Özel Duygan *et al*., 2020), are arguably also experiencing a resolution revolution. Overcoming the challenge of integrating data across these platforms will certainly promote a more integrative understanding of the evolutionary, ecological and physiological mechanisms that underpin microbial community structure, function and dynamics. With computational and experimental microbiomics advancing hand in hand, we will keep paving solid grounds to accurately predict the outcome of targeted modulations of the microbiome and to engineer its desired functional properties (Cullen *et al*. 2020; Widder *et al*. 2016). To foster the impact of such developments and the resultant translational applications, we advocate concomitant efforts into improving microbial literacy across all levels of society. This way, a more prominent role for microbiomics‐informed decisions in policy making could become more than just wishful thinking.
